# Multihospital Outbreak of *Clostridium difficile* Infection, Cleveland, Ohio, USA

**DOI:** 10.3201/eid1605.071606

**Published:** 2010-05

**Authors:** Robin L.P. Jump, Michelle M. Riggs, Ajay K. Sethi, Michael J. Pultz, Tracie Ellis-Reid, William Riebel, Dale N. Gerding, Robert A. Salata, Curtis J. Donskey

**Affiliations:** University Hospitals of Cleveland, Cleveland, Ohio, USA (R.L.P. Jump, R.A. Salata); Cleveland Veterans Affairs Medical Center, Cleveland (M.M. Riggs, M.J. Pultz, T. Ellis-Reid, C.J. Donskey); Case Western Reserve University, Cleveland (A.K. Sethi); Lakewood Hospital, Lakewood, Ohio, USA (W. Riebel); Hines Veterans Affairs Hospital, Hines, Illinois, USA (D.N. Gerding); Loyola University Chicago Stritch School of Medicine, Maywood, Illinois, USA (D.N. Gerding)

**Keywords:** Clostridium difficile, bacteria, outbreak, fluoroquinolone, formulary, expedited, Ohio, USA, dispatch

## Abstract

To determine whether a multihospital *Clostridium difficile* outbreak was associated with epidemic strains and whether use of particular fluoroquinolones was associated with increased infection rates, we cultured feces from *C. difficile*–infected patients. Use of fluoroquionolones with enhanced antianaerobic activity was not associated with increased infection rates.

Recent outbreaks of *Clostridium difficile* infection have been attributed to the emergence of an epidemic strain characterized as North American pulsed-field gel electrophoresis type 1 (NAP1) or restriction endonuclease assay group BI (*1**,**2*). Fluoroquinolone resistance is a hallmark of epidemic *C. difficile* isolates (*1*), and fluoroquinolone use has been associated with *C. difficile* infection (*2**–**9*). Because the C-8 methoxy fluoroquinolones gatifloxacin and moxifloxacin have enhanced antianaerobic activity, they might promote *C. difficile* infection to a greater degree than ciprofloxacin and levofloxacin (*10*). In 3 studies, substitution of gatifloxacin or moxifloxacin for levofloxacin was associated with an increase in *C. difficile* infection (*6**,**8**,**9*); in 2 of the 3 studies, a formulary change back to levofloxacin was associated with reduced *C. difficile* infection (*6**,**9*). However, ciprofloxacin and levofloxacin also have been associated with *C. difficile* infection (*2**–**5**,**7*).

Beginning in 2002, outbreaks of *C. difficile* infection occurred in several hospitals in the Cleveland, Ohio, USA, area. In response, the Ohio Department of Health (ODH) made *C. difficile* infection a reportable disease in 2006. One objective of the current study was to examine the magnitude of the outbreaks in Cuyahoga County, which comprises Cleveland and the surrounding area, and to determine whether the outbreaks were associated with epidemic BI/NAP1strains. A second objective was to examine whether use of gatifloxacin and/or moxifloxacin was associated with increased rates of *C. difficile* infection in healthcare facilities and to assess whether outbreaks correlated with formulary changes in fluoroquinolones.

## The Study

We used the ODH website (www.odh.state.oh.us) to obtain rates (cases/10,000 patient-days) of initial *C. difficile* infections during January–December 2006 for the 22 hospitals in Cuyahoga County. All healthcare facilities in Ohio were required to submit *C. difficile* infection rates by using a standardized method of reporting. An initial case was defined as a first positive laboratory diagnostic test for *C. difficile*, pseudomembranes on endoscopy, or confirmatory histologic features from surgical or autopsy specimen. An infection that occurred >6 months after a previous infection was classified as an initial infection.

For a subset of 5 hospitals, up to 20 consecutive stool samples from individual patients with *C. difficile* infection were cultured for *C. difficile* (*11*). *C. difficile* isolates were tested for in vitro cytotoxin production and moxifloxacin susceptibility and analyzed for binary toxin gene *cdtB* and partial deletions of the *tcdC* gene (*11**–**13*). Molecular typing was performed by using PCR ribotyping (*11*). The 5 hospitals were 1 community hospital, 3 tertiary care facilities, and 1 Veterans Affairs hospital. Three of the hospitals had experienced large outbreaks of *C. difficile* infection in 2002–2003 (i.e., their *C. difficile* incidence doubled and their peak incidence was >20 cases per 1,000 discharges); the other 2 reported an increase in the proportion of cases that were fulminant. The infection control departments of each institution provided information about *C. difficile* infection rates, fluoroquinolones on formulary, and infection control measures for *C. difficile* during January 2000–December 2006.

Rates of *C. difficile* infection for 2006 were compared among hospitals with moxifloxacin or gatifloxacin versus those with levofloxacin on formulary as primarily fluoroquinolones used to treat respiratory infections. In addition, for 2 hospitals in the molecular typing analysis that had a formulary change from 1 respiratory fluoroquinolone to another, we used Poisson analysis to compare rates of *C. difficile* infection during the 12 months before and after the formulary change, with a lag of 1 month after the change. We analyzed data using SPSS statistical software version 10.0 (SPSS Inc., Chicago, IL, USA) and STATA 9.1 (StataCorp, College Station, TX, USA).

For the 18 adult acute-care hospitals and 4 long-term acute-care (LTAC) facilities in Cuyahoga County, the median *C. difficile* infection rate in 2006 was 7.3 (range 4.2–63.1 cases/10,000 patient-days). The highest rates were observed in 2 LTAC facilities. Six facilities (3 acute care hospitals and 3 LTACs) had higher *C. difficile* infection rates than did each of the 5 hospitals in the molecular typing analysis.

A total of 64 toxigenic *C. difficile* isolates were cultured from feces samples obtained from 5 hospitals. Features of 42 (66%) isolates were consistent with epidemic BI/NAP1 strains (range 55%–83% for each facility), including amplification of the binary toxin gene *cdtB* and partial deletions in *tcdC* and resistance to moxifloxacin (MICs >32 µg/mL). By PCR ribotyping, we observed a characteristic banding pattern for isolates with features of the epidemic strain; 6 isolates with this banding pattern were confirmed as BI strains in the laboratory of D.G.

Of the 22 facilities, 8 used moxifloxacin as the primary respiratory fluoroquinolone, 13 used levofloxacin, and 1 did not have a respiratory fluoroquinolone on formulary. The *C. difficile* infection rate did not differ between facilities with levofloxacin (8.5 cases/10,000 patient-days, 95% confidence interval [CI] 7.8–9.3) and moxifloxacin (8.5 cases/10,000 patient-days, 95% CI 7.8–9.2) on formulary (p = 1) (Table). The facility that did not have a respiratory fluoroquinolone on formulary had a lower rate of *C. difficile* infection than the median rates for facilities that used levofloxacin or moxifloxacin. However, 8 facilities had lower *C. difficile* infection rates than did this institution.

**Table:** *Clostridium difficile* infection rates and healthcare facility characteristics according to respiratory fluoroquinolone on formulary, Cleveland, Ohio, USA, 2006*

Characteristic	Levofloxacin	Moxifloxacin	Neither†	Total
No. hospitals	13	8	1	22
No. beds, median (IQR)	232 (108–371)	361 (217–565)	1,008	316 (125–424)
Type of facility				
Tertiary care	0	2	1	3
Acute care	9	6	0	15
Long-term acute care	4	0	0	4
Hospital system				
System 1	1	5	0	6
System 2	11	0	1	12
Neither	1	3	0	4
No. cases of *C. difficile* infection	494	569	206	1,269
Patient-days	580,893	666,719	293,833	1,541,445
Rate of *C. difficile* infection/10,000 patient-days, median (IQR)	8.5 (7.8–9.3)	8.5 (7.8–9.2)	7.0 (6.1–8.0)	8.2 (7.8–8.7)

*IQR, interquartile range. †Ciprofloxacin on formulary but no respiratory fluoroquinolone on formulary.

Two of the 5 hospitals in the molecular typing analysis changed their formulary fluoroquinolones during the study period (Figure). Both hospitals made formulary changes from levofloxacin to gatifloxacin; however, the increase in *C. difficile* infection rates preceded the formulary change in each hospital. *C. difficile* infection rates did not differ significantly in the 12 months before and after the change from levofloxacin to gatifloxacin (relative risk [RR] 1.0, 95% CI 0.97–0.86; p = 0.973). For hospital 2 (Figure, panel B), a subsequent formulary change from gatifloxacin to levofloxacin was associated with a reduction in *C. difficile* infection (RR 0.59, 95% CI 0.51–0.70; p<0.001); an intervention to improve environmental cleaning with a 10% bleach solution occurred at the time of the formulary change.

**Figure:**
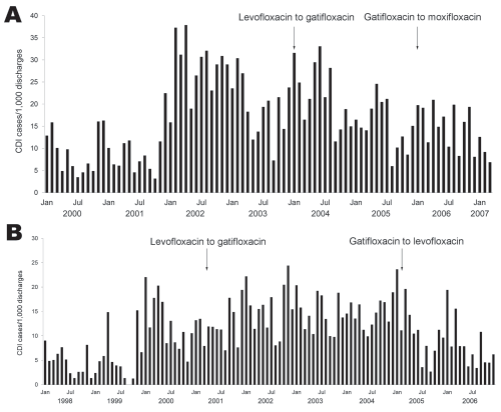
Rates of *Clostridium difficile* infection for hospital 1 (A) and hospital 2 (B). Arrows indicate the timing of the formulary changes in fluoroquinolone antimicrobial drugs.

## Conclusions

Our findings provide further evidence that emergence of epidemic NAP1/BI strains in a geographic region may be associated with large multihospital outbreaks of *C. difficile* infection. Before the ODH decision to require mandatory reporting, many area hospitals were either not collecting surveillance data about *C. difficile* infection or were reluctant to share their rates. Therefore, we believe that mandatory public reporting of *C. difficile* infection rates provided a valuable tool to examine the full magnitude of the outbreaks and an incentive for hospitals with high rates to increase efforts to control infection. One area hospital recently reported that the ODH database underestimated the incidence of *C. difficile* infection (*14*), but this observation does not affect our conclusions because all facilities used the same surveillance definitions. Our findings do not support the hypothesis that use of moxifloxacin or gatifloxacin is associated with higher rates of *C. difficile* infection than is use of levofloxacin or ciprofloxacin.

Our analysis of formulary fluoroquinolones and *C. difficile* infection has several limitations. First, data on the amount of the fluoroquinolones used in the hospitals were not available. Second, analysis of hospital formularies does not account for the effects of fluoroquinolones used in long-term care facilities and among outpatients. Third, we did not assess confounding factors, such as use of other classes of antimicrobial drugs and differing patient populations. Finally, studies that evaluate group-level effects may not reflect the biological effects at the individual-patient level. Additional studies are needed to evaluate the risk for *C. difficile* infection associated with different fluoroquinolones.
